# A Simple Spectrophotometric Method for the Determination of Thiobarbituric Acid Reactive Substances in Fried Fast Foods

**DOI:** 10.1155/2016/9412767

**Published:** 2016-03-31

**Authors:** Alam Zeb, Fareed Ullah

**Affiliations:** Department of Biotechnology, University of Malakand, Chakdara 18800, Pakistan

## Abstract

A simple and highly sensitive spectrophotometric method was developed for the determination of thiobarbituric acid reactive substances (TBARS) as a marker for lipid peroxidation in fried fast foods. The method uses the reaction of malondialdehyde (MDA) and TBA in the glacial acetic acid medium. The method was precise, sensitive, and highly reproducible for quantitative determination of TBARS. The precision of extractions and analytical procedure was very high as compared to the reported methods. The method was used to determine the TBARS contents in the fried fast foods such as Shami kebab, samosa, fried bread, and potato chips. Shami kebab, samosa, and potato chips have higher amount of TBARS in glacial acetic acid-water extraction system than their corresponding pure glacial acetic acid and vice versa in fried bread samples. The method can successfully be used for the determination of TBARS in other food matrices, especially in quality control of food industries.

## 1. Introduction

Food frying is one of the oldest known procedures in human history. Frying is carried out using oils or fats. The major composition of these frying mediums is triacylglycerols. During frying triacylglycerols and fatty acids are oxidized to form primary oxidation products [1]. These products include hydroperoxides, epoxides, epidioxides, hydroxides, and several other products with simple or complex structures [2, 3]. Recent studies showed that several analytical methods can be applied to determine their absolute structures [4, 5]. However, due to the complex nature of food frying, it is still debatable to conclude on each specific method or oxidized compounds produced during frying. It was found that the primary oxidation products are further oxidized to form secondary and tertiary oxidation products. These products include a high amount of aldehydes with small to large chain structures [6]. The aldehydes and other reactive substances are one of the main causes of rancidity in foods during preparation and storage [7]. One of the important oxidation products is known as malondialdehyde (MDA), which is considered as the main marker in lipid peroxidation. Thiobarbituric acid (TBA) is reacted with MDA, which is resulting in a colour compound, which can be determined spectrophotometrically, chromatographically, or through image processing techniques [8–10]. Due to the reactivity of TBA with several reactive substances in the biological sample, a more widely accepted terminology called thiobarbituric acid reactive substances (TBARS) is now commonly used [11].

TBARS is now considered as a standard marker for the lipid peroxidation induced oxidative stress [12]. Meat or meat products upon frying also produced several oxidation products, which can be measured using TBA-MDA adduct with the help of HPLC. Several HPLC methods are available regarding the TBARS assay [9, 13]. However, due to the high cost and long operation timing of the HPLC procedures, a more simple method is therefore required. Botsoglou et al. [14] developed a spectrometric method for the TBARS as marker for lipid oxidation in animal tissues, food, and feed substances. However, the method involves 1,1,3,3-tetraethoxypropane (TEP) as MDA precursor, the hydrolysis of which would need a trained analytical chemist. The method also involves hexane during extraction procedures, which was found to cause losses in the MDA contents from the fried fast foods and was thus not applicable for fried fast foods. Recently Papastergiadis et al. [15] developed a spectrophotometric method for analysis of MDA in oxidized foods such as peanuts, almonds, walnuts, cookies, crisps, and trout samples. Their method was comparable with HPLC method; however, there is a lack of information about the MDA analysis in complex fried food matrix such as traditional fried street foods. This paper presents a simple and highly sensitive spectrometric method for the analysis of TBA reactive substances in the traditional fried street foods.

## 2. Experimental

### 2.1. Chemicals and Reagents

Thiobarbituric acid (TBA) 99% pure was purchased from BDH (BDH, England); malondialdehyde tetrabutylammonium salt (MDA salt) 96% pure and methanol 99.8% pure were from Sigma-Aldrich (Steinheim, Germany). Glacial acetic acid (99–101% pure) was purchased from Daejung (Daejung, Korea). Ultrapure deionized double distilled water with less than 5 mΩ was used. All other chemicals and reagents were of an analytical standard with high purity.

### 2.2. Preparation of TBA Reagent

The standard solution of 4.0 mM of TBA was prepared in glacial acetic acid. For this purpose, 57.66 mg of TBA was dissolved in 100 mL of glacial acetic acid. Fresh solution of TBA was prepared every day.

### 2.3. Preparation of MDA and Calibration Standards

Standard stock solution of MDA (1 mM) was prepared in glacial acetic acid. MDA (31.35 mg) was accurately weighed and dissolved in 100 mL solvent. From the stock solution, different concentrations of 0.1, 0.2, 0.4, 0.6, and 0.8 mM were prepared. The calibration curve was constructed in the concentration range of 0.1 to 1.0 mM.

### 2.4. Extraction of TBARS in Fried Samples

One gram of each fried grinded sample (samosa, Shami kebab, fried bread, and potato chips) was taken in 25 mL test tube and 5 mL of the solvent. The solvent was either 100% glacial acetic acid (AA) or 50% glacial acetic acid in water (AW). BHT (0.01%) was used to prevent further oxidation of the medium. The samples were shaken for 1 h and filtered. The filtrate was centrifuged, when required, and was used for analyses.

### 2.5. Analytical Procedure

The standard MDA solution (1 mL) was taken in a 10 mL test tube and mixed with TBA (1 mL). The mixture was heated in a boiling water bath at 95°C for 60 minutes. The test tubes were cooled at room temperature and absorbance was measured at 532 nm using UV-visible spectrophotometer model PharmaSpec 1700 (Shimadzu, Japan). Each standard for the calibration was repeated (*n* = 3) according to the above procedure. A blank sample was repeated (*n* = 5) replacing standard or sample by acetic acid or water.

Samples of the fast foods collected were samosa, Shami kebab, fried bread, and potato chips. Two different kinds of samples extracts were prepared, that is, with 100% glacial acetic acid (AA) and 50% glacial acetic acid with water (AW). The extract of each sample (1 mL) was mixed with 1 mL TBA reagent and the above procedure was repeated five times (*n* = 5). The TBARS was calculated using the formula as *μ*M/g of the sample:

(1) $$\begin{array}{c} TBARS\;\;\left(\;\mu M/g\;\right)=\left(\;Ac\times V\;\right)/W, \end{array}$$

where Ac is the amount determined from the calibration curve and *W* is the weight of the sample taken while *V* is volume in mL or dilution factor of the total extract prepared.

### 2.6. Method Validation

The analytical method was validated according to the guidelines of the International Conference on Harmonization (ICH). Linearity was determined from the different concentration measured (*n* = 3) in the range of 0.1–1.0 *μ*M. Limits of detection (LOD) and limits of quantitation (LOQ) were determined from the standard calibration curve. Precision was determined in terms of intraday (*n* = 3) and interday (*n* = 5) at three concentration levels of 0.1, 0.4, and 0.8 *μ*M. Stability was measured (*n* = 9) at concentration of 0.8 *μ*M. The accuracy of the method was measured using recovery studies in samosa samples (*n* = 9) at two different extraction procedures. The pure glacial acetic acid has extraction code of AA, while AW was code given to 50% glacial acetic acid in water.

## 3. Results and Discussion

### 3.1. Optimization of Analytical Conditions

**Figure 1:**
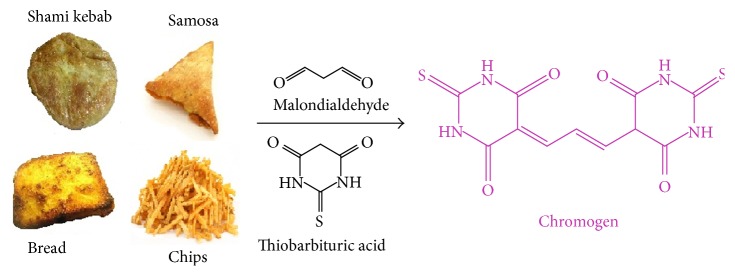
Typical reaction mechanism of malondialdehyde (MDA) and thiobarbituric acid (TBA) from the fried fast foods. The reaction was carried out at 95°C in water bath for 60 minutes. The pink colour complex is measured spectrophotometrically at 532 nm.

Different analytical conditions were evaluated for extraction and preparation. Glacial acetic acid was found to be the best solvent for extractions and preparation of reagent. Botsoglou et al. [14] used different quantity of trichloroacetic acid for the preparation of standard reagents and extractions. The present method is based on the unified solvent system with high dissolution and extraction power. TEP used in the previous studies [14, 16] has been found to produce artifacts and therefore MDA salt was used in this study. This study thus does not require preparation of buffers needed for the hydrolysis of TEP and also does not produce the false negative results from its partial hydrolysis. The standard MDA salt gives a high accurate standard curve and stable spectrophotometer readings. The MDA-TBA mixture was reacted for 60 min (Figure 1). Previous study and our observations showed that 60 min was optimum time for MDA-TBA reaction [14]. Two extractions were performed using glacial acetic acid (100%) and glacial acetic acid (50%) in water.

### 3.2. Method Validation

**Figure 2:**
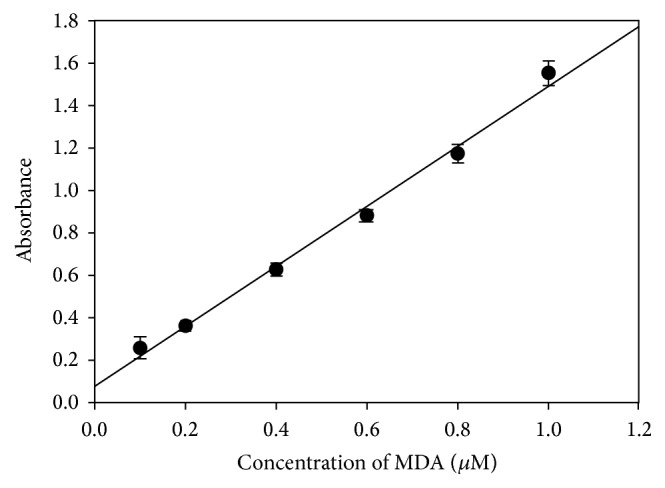
A linear regression curve of standard concentration of 0.1–1.0 *μ*M with a correlation coefficient of 0.9929 and regression equation of *y* = 1.4167*x* + 0.0785. Each point in the regression represents the replicate measurement (*n* = 3).

The developed method was validated for its quantitative performance using a standard calibration curve. The calibration curve of six points in triplicate (*n* = 3) was established in the concentration range of 0.1 to 1.0 *μ*M. The present method was more sensitive than previously reported by Papastergiadis et al. [15], who showed a linearity range of 0.6–10 *μ*M. Linear regression shows a correlation coefficient of 0.9929 with equation of *y* = 1.4167*x* + 0.0785 as shown in Figure 2. The LOD and LOQ were evaluated from the slope and residual standard deviations of the standard curve. The LOD was 1.758 *μ*M, while LOQ was 5.859 *μ*M. Instrumental precision was determined by replicate (*n* = 9) analysis of standard compounds. The results showed higher precision (4.23, % RSD) for pure glacial acetic acid than 50% glacial acetic acid (6.37, % RSD). This shows that pure glacial acetic acid was a better solvent.

Repeatability (% RSD) was determined using intraday and interday analyses of three standard concentrations (0.1, 0.4, and 0.8 *μ*M) in replicates (*n* = 3). The intraday precision for the concentration of 0.1 *μ*M was 11.3%, that for concentration of 0.4 *μ*M was 10.8%, and that for concentration of 0.8 *μ*M was 2.03% (% RSD). This shows that the precision was higher at high concentration. The interday precisions of 8.4, 7.9, and 13.1% were obtained for the concentration of 0.1, 0.4, and 0.8 *μ*M, respectively, as shown in Table 1. The overall precision of this method was higher than reported methods [14, 15, 17]. The specificity of the method was assessed using the absorption spectra of the standard MDA-TBA adduct formed after reaction and also in the sample. It was found that absorption spectra were a good tool for determination of specificity of individual class of compound.

The accuracy of the method was evaluated using recovery studies. Samples of samosa were spiked with 0.4 *μ*M of the standard addition method (*n* = 9). The recovery studies were carried out in both pure glacial acetic acid and 50% glacial acetic acid. Results show a higher recovery for pure glacial acetic acid samples (114.3%) than its corresponding 50% glacial acetic acid samples (96.01%). The recovery of the method was higher than other spectrophotometric methods reported previously [15, 18]. The extraction using pure glacial acetic acid was found to have higher recovery than previously reported methods with HPLC as shown in Table 2 [10, 17].

### 3.3. TBARS in Fried Foods

Four samples of the street foods were selected, because of the wide uses and popularity. These include Shami kebab, samosa, fried bread, and potato chips. The short description of these foods is given in Table 3. Shami kebab is made of cooked grinded chickpeas, hot spices, onion, garlic extract, and grinded tomatoes and was fried in vegetable ghee. The AA extract has lower TBARS values (1.10 ± 0.06 *μ*M/g, mean ± SD) than its corresponding AW extracts (1.505 ± 0.09, mean ± SD). Pandey et al. [19] revealed recently that deep fried Shami kebab had higher amount of TBARS than the grilled one and reported lower values of TBARS than the present studies. The difference may be due to the difference in the laboratory and fast food restaurant frying or frying medium. These results indicate that the present simple method can give us a good look at the check and balance system in the fast food restaurants or streets foods frying.

The typical composition of samosa consists of wheat starch, onion, potatoes, and tomatoes. Only the outer crispy part of the samosa was analyzed, because this part is highly exposed to frying temperature and frying oil. Similar to Shami kebab, the lower amount of TBARS was obtained in AA samples (1.02 ± 0.18 *μ*M/g, mean ± SD) than its corresponding AW samples (2.51 ± 0.19 *μ*M/g, mean ± SD). Fried bread has a typical composition of wheat bread mixed with eggs and milk. A higher amount of TBARS was obtained in AA samples (0.891 ± 0.09 *μ*M/g, mean ± SD) than its corresponding AW samples (0.372 ± 0.03 *μ*M/g, mean ± SD). The difference in the TBARS may be due to its dissolution power and polarity of the solvent and solute.

The results of the TBARS of the potato chips showed a higher amount in AW (2.911 ± 0.13 *μ*M/g, mean ± SD) than AA (2.21 ± 0.13 *μ*M/g, mean ± SD). Potato chips are generally consumed fresh or later after preparation. The high amounts of lipids, high surface-to-volume ratio, and packaging in the presence of oxygen can result in oxidative deterioration of the chips [20]. Thus, it is imperative to keep samples under standard conditions, while checking the proper healthy level using simple laboratory protocol.

## 4. Conclusions

In this study, a simple and sensitive spectrophotometric method was developed for the determination of TBARS in fried street foods. The method uses glacial acetic acid for the preparation of standards and samples. The method was precise, sensitive, and highly reproducible for quantitative determination of TBARS. The precision of extractions and analytical procedure was very high as compared to the reported methods. The method was used to determine the TBARS contents in the fried foods such as Shami kebab, samosa, fried bread, and potato chips. The method can be successfully used for the determination of TBARS in other food matrices for quality control analysis in food industries or regular food inspection system.

## Figures and Tables

**Table 1:** Intraday and interday precision of the spectrophotometric TBARS method.

Concentration	Mean concentration (*µ*M/g)									
	Intraday precision (*n* = 3)					Interday precision (*n* = 5)				
	Morning	Noon	Evening	Mean	% RSD	Day 1	Day 2	Day 3	Mean	% RSD
0.1	0.292	0.325	0.366	0.327	11.3	0.353	0.325	0.385	0.354	8.4
0.4	0.629	0.545	0.677	0.617	10.8	0.629	0.677	0.737	0.681	7.9
0.8	1.174	0.926	0.956	0.948	2.03	1.172	0.926	0.962	1.02	13.1

**Table 2:** Recovery (*n* = 9) and stability (*n* = 9) studies of the spectrophotometric method.

Extraction type	Extraction code	Amount already present (*µ*M/g)	Amount added (*µ*M/g)	Theoretical total (*µ*M/g)	Recovered amount (*µ*M/g)	Recovery (%)	Stability % RSD
Glacial acetic acid (100%)	AA	1.02 ± 0.18	0.4	1.42	1.62 ± 0.21	114.3	4.23
Acetic acid (50%) in water	AW	2.51 ± 0.19	0.4	2.91	2.79 ± 0.28	96.01	6.37

**Table 3:** Thiobarbituric acid reactive substances (TBARS) in fried fast foods samples. AA is 100% acetic acid, while AW is 50% acetic acid in water.

Sample name	Description		TBARS (*µ*M/g) *n* = 5	
	Composition	Frying medium	AA	AW
Shami kebab	Cooked grinded chickpeas, hot spices, onion, garlic extract, grinded tomatoes	Vegetable ghee	1.10 ± 0.06	1.505 ± 0.09
Samosa	Wheat starch, onion, potatoes, tomatoes	Vegetable oil	1.02 ± 0.18	2.51 ± 0.19
Bread fried	Wheat bread, eggs, milk	Vegetable ghee	0.891 ± 0.09	0.372 ± 0.03
Potato chips	Potato chips freshly prepared	Vegetable oil	2.21 ± 0.13	2.911 ± 0.13
